# Identifying roles in smoking cessation care for different types of healthcare providers: a qualitative study with people who smoke

**DOI:** 10.3389/frhs.2025.1421429

**Published:** 2025-04-14

**Authors:** Naomi A. van Westen-Lagerweij, Elisabeth G. Meeuwsen, Esther A. Croes, Niels H. Chavannes, Eline Meijer

**Affiliations:** ^1^ The Netherlands Expertise Centre for Tobacco Control, Trimbos Institute, Utrecht, Netherlands; ^2^ Department of Public Health and Primary Care, Leiden University Medical Center, Leiden, Netherlands

**Keywords:** smoking, healthcare, focus groups, lower socio-economic groups, perceptions, smoking cessation care, professional roles

## Abstract

**Objective:**

This study aimed to explore how people who smoke, in particular those with a lower socioeconomic position, perceive the roles of different healthcare providers in smoking cessation care.

**Methods:**

Three semi-structured focus groups were held with a total of 15 people who smoke in community centres situated in low socioeconomic position neighbourhoods in the Netherlands. The focus groups were part of a larger study aimed at improving the delivery of smoking cessation care within primary care. Focus group transcripts were thematically analysed.

**Results:**

Participants believed it is the role of the general practitioner to initiate a conversation about smoking and inform them about the options for quitting. A quit advice from a medical specialist carried most weight for several participants. Participants felt that pharmacists and doctor's assistants are insufficiently qualified to advise on quitting smoking.

**Conclusion:**

People who smoke and have a lower socioeconomic position seem to have clear ideas about the roles of different healthcare providers in smoking cessation care. These ideas appear to be tied to the perception of whether a healthcare provider is qualified to address smoking.

**Practice implications:**

Doctors can use their authority to address smoking and provide advice. Actions are needed to improve people's perceptions of non-physicians in smoking cessation care.

## 1 Introduction

Tobacco smoking remains a serious public health threat worldwide (1), with the prevalence of smoking being highest among lower socioeconomic position (SEP) groups (i.e., people with low educational attainment and/or low income, and as a result are often faced with poverty and poor health) (2). Socioeconomic inequalities in the prevalence of smoking are, in part, the result of inequalities at various points along the journey to quitting smoking. Compared to their higher-SEP counterparts, lower-SEP people who smoke are less likely to intend to quit, make a quit attempt, and successfully quit smoking (3). An important explanation for these differences is that lower-SEP people who smoke face many barriers in accessing evidence-based cessation support, such as low social support, competing priorities, lack of knowledge, and negative attitudes towards support (4). A lack of access to evidence-based cessation support means that many lower-SEP people who smoke are not receiving the help which they may need to successfully quit smoking.

Healthcare providers can play an important role in addressing smoking among lower-SEP people who smoke, motivating them to quit smoking, and offering them, or referring them to, evidence-based cessation support. However, studies have shown that not all healthcare providers believe they can play an important role in helping people who smoke quit or that it is their responsibility to address smoking (5, 6). In addition, healthcare providers do not always know who is responsible for smoking cessation care (7). Previous research demonstrated that role identity (i.e., the perception that smoking cessation care is part of a professional's role) strongly predicts whether healthcare providers address smoking and refer to cessation support (8).

So far, the role of healthcare providers has mostly been studied from the perspective of healthcare providers themselves. Little is still known about the perceptions of (lower-SEP) people who smoke regarding the role of healthcare providers. A previous Dutch study found that primary care providers, and in particular general practitioners (GPs), are an important source for lower-SEP people who smoke to both hear about and be referred to smoking cessation counselling (9). These findings were, however, based on people who had recently received smoking cessation counselling, meaning that the group most likely consisted of people who wanted help with quitting smoking. The reality is that many people who smoke do not want any assistance with quitting smoking and prefer to quit on their own (10, 11). People who smoke may strongly believe quitting is their personal responsibility and that quitting without professional help is the right thing to do (10). Thus, the question arises: according to people who smoke, what role can healthcare providers play in the journey to quitting smoking, especially when the people who smoke perceive little need for smoking cessation support?

In the current study we explored views on the roles of different healthcare providers in smoking cessation care among a diverse group of people who smoke. The group consisted of 15 individuals, both those who wanted professional help to quit smoking and those who did not. We define “smoking cessation care” as all steps a healthcare provide can take to help someone quit smoking, including asking about tobacco use, advising and motivating to quit, and offering or referring to cessation support. We primarily recruited lower-SEP people who smoke. People who had quit smoking in the past five years were also recruited, as their perceptions are relevant as well. Knowledge about the potential role of healthcare providers, from the perspective of people who currently smoke and those who have quit, may eventually help to identify opportunities and barriers for smoking cessation care among lower-SEP people who smoke.

## 2 Methods

### 2.1 Study design and participants

In this qualitative study we conducted three focus groups with people who currently smoke and people who have quit smoking in the Netherlands. The focus groups were part of a larger study, aimed at improving the delivery of smoking cessation care within primary care (in this case: general practice and pharmacy) in the Netherlands. The goal of the focus groups was to explore the views of people who currently smoke and people who have quit smoking regarding smoking cessation care, with a focus on primary care. An advantage of focus groups, compared to individual interviews, is that they allow interaction between participants, which can help to clearly identify agreement and disagreement within a group (12). We aimed for five to eight participants per focus group, as recommended in the literature (13). Larger groups are not ideal, as they are more difficult to control and limit each participant's opportunity to share ideas.

Participants were eligible if they currently smoked and (maybe) wanted to quit smoking, or if they had quit smoking in the past five years. Although no inclusion criterion was formulated for SEP, we used targeted recruitment strategies to identify and recruit mostly lower-SEP participants. Since it can be challenging to recruit lower-SEP people in research, we used a personal approach by recruiting people in their own communities (14). Participants were recruited from three different community centres situated in low socioeconomic position neighbourhoods in the cities of Utrecht and Leiden. We first contacted staff from the community centres, and asked them to help us with personally approaching people who currently smoke and people who have quit smoking at the community centres. Since we aimed to mostly recruit lower-SEP participants, we approached people during daytime, as people who are unemployed and/or receive social assistance benefits typically visit the community centres during daytime. With this personal approach we managed to recruit twelve participants. We also distributed flyers in the neighbourhoods of the community centres, and posted recruitment messages on social media, and managed to additionally recruit three more participants.

### 2.2 Procedure

The focus groups were conducted in May and November 2019 in the three different community centres (i.e., one focus group per community centre). Before participation, all participants received written information about the study and confidentiality procedures. Participants were informed that participation is voluntary and that they can withdraw from the study at any time. Participants each received 15 euros to thank them for study participation.

Two focus groups were led by the authors NAvW-L and EGM (one person moderated the focus group, while the other made notes), and one focus group was led by EGM and a trained master student (EGM moderated the focus group, while the master student made notes). All three had a background in health policy or medicine, and had no relationship with the participants prior to the study. NAvW-L and EGM both had experience with conducting qualitative research, which helped with moderating the focus groups as well as later on analysing the results.

Before the start of the focus groups, participants were asked to sign an informed consent form and to complete a short questionnaire with questions regarding their demographics, such as age, highest level of education completed (which is an indicator of SEP), and experience with quitting smoking. Focus groups were audio recorded and lasted around 90 min, which gave the interviewers enough time to discuss all topics without burdening the participants too much. A semi-structured focus group guide was used to guide the conversation (see the Supplementary Material for the questions).

### 2.3 Ethics

This study was conducted in line with the guidelines of the Helsinki Declaration of Good Clinical Research Practice, and was approved by the Trimbos Institutional Ethics committee.

### 2.4 Analysis

The focus groups were transcribed verbatim. Qualitative content analysis was conducted using the software package ATLAS.ti. EGM and the master student first coded one randomly selected focus group independently from each other. Using the topics from the focus group guide, they applied thematic coding. In addition, they applied open coding to capture relevant data. They then discussed the codes and agreed upon any new codes and categories, after which the master student coded the remaining transcripts. Disagreements were resolved through discussion between EGM and the master student, consulting EM in cases where disagreements remained unresolved after discussion between EGM and the master student. After coding the first two transcripts, no new codes emerged in the third transcript, indicating that data saturation was reached. NAvW-L used the codes to identify important categories and themes for this study. First, categories were formed by identifying similarities in codes across the focus groups and addressing questions such as *who*, *what* and *where* (14). Next, within each category, themes were identified by addressing questions such as *why*, *how*, and *in what way* (14). In this stage of interpretation, similarities and differences between focus groups were analysed. The identified categories and themes were continuously compared against the transcripts and adjusted if necessary.

The quotes presented in this study were translated by the first author from Dutch to English. In the quotes, the interviewer is indicated by the letter “I”. Each participant is identified by a number (1–15), and the letter “S” in front of the number indicates they currently smoked, while the letter “F” indicates they formerly smoked.

## 3 Results

Table 1 presents participant characteristics. Fifteen people participated, of whom 12 currently smoked (80%) and three formerly smoked (20%). The participants who formerly smoked had quit smoking between one month and two years ago. All participants smoked daily, or used to smoke on a daily basis before quitting smoking. Seven participants smoked or used to smoke cigarettes (on average 16 per day), seven participants smoked or used to smoke rolling tobacco (on average two packages per week), and one participant smoked cigarillos (10 per day). Most participants were female (67%) and had attained a low level of education (67%). Only three participants had a paid job (all three with a medium or high level of education). On average, participants were 54 years old and had attempted to quit smoking four times. Only three participants (of which one who formerly smoked) had received professional help during a quit attempt in the past: one from a practice nurse specialised in mental health care, one from a general practitioner, and one from a pulmonary nurse.

**Table 1:** Characteristics of the participants.

Characteristics	Total (*n* = 15)	Group A (*n* = 5)	Group B (*n* = 5)	Group C (*n* = 5)
Smoking status—*n* (%)				
Currently smoking	12 (80)	4 (80)	5 (100)	3 (60)
Formerly smoking	3 (20)	1 (20)	0 (0)	2 (40)
Gender—*n* (%)				
Male	5 (33)	2 (40)	1 (20)	2 (40)
Female	10 (67)	3 (60)	4 (80)	3 (60)
Age (in years)				
Mean (SD)	54 (14)	50 (12)	52 (20)	59 (9)
Educational attainment^a^—*n* (%)				
Low	10 (67)	4 (80)	2 (40)	3 (60)
Medium	3 (20)	0 (0)	2 (40)	1 (20)
High	2 (13)	1 (20)	1 (20)	0 (0)
Paid job—*n* (%)				
Yes	3 (20)	1 (20)	2 (40)	0 (0)
No	12 (80)	4 (80)	3 (60)	5 (100)
Type of tobacco product used—*n* (%)				
Cigarette	7 (47)	1 (20)	2 (40)	4 (80)
Rolling tobacco	7 (47)	3 (60)	3 (60)	1 (20)
Other	1 (7)	1 (20)	0 (0)	0 (0)
Tobacco consumption				
Mean no. of cigarettes per day among cigarette users (SD)	16 (20)	18 (0)	7 (5)	21 (26)
Mean no. of packages of rolling tobacco per week among rolling tobacco users (SD)	2 (1)	2 (0)	2 (2)	3 (0)
Number of previous quit attempts				
Mean (SD)	4 (5)	2 (1)	5 (8)	3 (3)
Received professional help during a quit attempt—*n* (%)				
Yes	3 (20)	2 (40)	1 (20)	0 (0)
No	10 (67)	3 (60)	3 (60)	4 (80)
Not applicable (has not attempted to quit before)	2 (13)	0 (0)	1 (20)	1 (20)

^a^ For “educational attainment” we used the highest level of education completed. “Low” corresponds with elementary school, lower secondary education or lower vocational education; “medium” corresponds with intermediate vocational education or higher secondary education; “high” corresponds with higher vocational education or university.

Regarding the need for professional help to quit smoking among those who currently smoked, five participants indicated that they would like to receive professional help during a quit attempt. Participants mentioned different reasons for wanting professional help, such as not having anyone close to them who can support them, or finding it scary to quit by themselves.

S9: “I live by myself, and if you don't have anyone to look after you, you do need support.”

S5: “My father quit smoking at once, and became delirious. That’s why I find it scary to quit smoking at once, because I could become delirious too. I really need someone to help me with that.”

Five participants indicated that they do not want professional help with quitting smoking, and two participants would only want help if they would feel that they had no other choice.

S8: “As soon as I feel I need professional help, I become pathetic. I don’t want that. I’m not pathetic.” (…)

I: “You don’t want to appear pathetic?”

S8: “Not like a victim.”

S12: “I just think it’s weak to ask for help. I feel like it has worked for a lot of people, if you really put in the effort then it should be possible [to quit without help].”

S15: “I would really need to receive a death notice, an almost-death notice, like: ‘if you don't stop now, you'll die tomorrow’ or something like that. Then it really needs to happen.”

Regarding the perceived potential role of healthcare providers in smoking cessation care, two categories were identified: (1) perceptions of the role of the GP, and (2) perceptions of the role of other healthcare providers. Within each category different themes were identified. Regarding the perceived role of the GP, themes that emerged were: (1) why participants would contact the GP for help, (2) the tasks they see for the GP, and (3) their preferred way of being approached by the GP. Regarding the perceived role of other healthcare providers, the dominant theme was the perceived expertise and authority of healthcare providers (in this case: pharmacists, doctor’s assistants and medical specialists).

### 3.1 Perceptions of the role of the GP

When asked who they would first contact if they were to seek advice or help with quitting (even if they do not want help with quitting now), most participants mentioned that they would first contact their GP. One reason is that the GP can refer them to professional help. Participants in group A agreed that the GP is necessary for a referral:

F2: “I was very afraid of gaining weight when quitting smoking. So, for that reason, I approached the GP.”

I: “So the GP is a logical go-to person for you?”

F2: “Yes, for me, it is.”

I: “And why the GP?”

F2: “Because they could refer me to the practice nurse.”

I: “And for the others, who would be your first go-to person if you needed help to quit smoking?”

S4: “The GP.”

S5: “Yes, for me too. Yes, the GP knows the way, I think.”

S4: “I don't think you can get help anywhere without a referral from the GP.”

A few participants also mentioned that it is logical to go to the GP, and not for example a pharmacist, because they already have a relationship with the GP, as mentioned in group B:

S6: “I would rather talk to the GP.”

S9: “I also find the GP more appropriate than the pharmacist.”

S6: “You have more of a connection with the GP. It’s different with the pharmacist.”

S9: “Yes, you have a relationship of trust with [the GP]. And a pharmacy is just a store.”

S6: “The GP knows everything about you.”

With regard to smoking cessation care, it became clear that most participants saw two tasks for the GP. First, most participants saw it as part of the role of the GP to initiate a conversation about smoking. As mentioned in group C:

F14: “Yes, of course [the GP] can say that. If they ask ‘do you smoke?’, and you say ‘yes’, then they say ‘it’s better to quit’. Yes, I know that. But I wouldn't mind if they bring it up.”

I: “Why not?”

F14: “Because it’s [the GP’s] job to help you.”

This participant who formerly smoked perceived a quit advice as the GP's task, and as something that could help the patient. The other participants in group C agreed, but one participant (S15) remarked that it feels like nagging if the GP initiates a conversation about smoking during each consultation. In line with this, participants in group A felt that the GP can only bring up the subject of quitting smoking if there is a medical reason to do so.

S4: “If there’s nothing medically wrong and you're just living your life, (…) then I don't think they should start talking about quitting smoking. It’s none of their business.”

(…)

F2: “Yes, I agree. Unless there’s a medical reason.”

Second, with regard to the tasks of the GP, most participants mentioned that they would appreciate receiving information from the GP about options to quit smoking, after which they can choose an option themselves. This was even mentioned by participants who initially said they do not want professional help with quitting. Participants in group A said the following:

S5: “When I go to the doctor, and I want to quit smoking, the best thing is for them to say ‘well, you can do this, you can do that, or you can do that’.”

S4: “Provide information that you can read at home. (…) And websites with information to read, so you can make a decision for yourself and stand behind your decision.”

F2: “That’s exactly what the GP did for me. Provided me with options and some time to think about it. That way you are in control.”

Two participants in group C, however, mentioned that they would not find it relevant to receive information about cessation help because they would not be interested in it.

F14: “For me that is not important at all. I just think you need to [quit] by yourself.”

When the GP initiates a conversation about smoking or provides information about quitting, the approach of the GP appears to be crucial. Participants in group B noted that they would find it unpleasant if the GP judged them or told them what to do. According to the participants in group B, it is better when the GP asks open questions such as “do you want to quit smoking” or “do you know what the options are to quit”.

S8: “I would find it very annoying if the GP tells me what I can or cannot do. (…) But I do think it’s good that questions are asked.”

S10: “Yes.”

S8: “‘Do you smoke?’ (..) Or ‘have you ever thought about quitting?’. That’s an open question. And then I would appreciate it if you can say yes or no, without any further judgment. And also ‘did you know that there are such and such groups, did you know that there are support groups?’.”

### 3.2 Perceptions of the role of other healthcare providers

Other types of healthcare providers were also briefly discussed, such as pharmacists, doctor's assistants, and medical specialists. While discussing the different types of healthcare providers, it became clear that participants based their idea of healthcare providers' roles on their perception of whether a healthcare provider has enough expertise or authority to address smoking. For example, in all three groups, pharmacists were seen as not qualified enough to discuss smoking or provide advice about quitting.

F2: “I don't think [giving advice on quitting] is the task of the pharmacist.”

S5: “Yes, I agree. They are just a bit too low on the ladder of healthcare, so to speak.”

As already mentioned earlier, most participants viewed a pharmacy as “just a store”, and thus may have felt it is inappropriate for a store employee to provide smoking cessation care. Participants, however, did see it as the role of the pharmacist to provide information about medication for quitting smoking, such as nicotine patches, since that is what they perceived the pharmacist as trained to do. When asked whether the pharmacist can also address smoking when someone picks up birth control pills or a pregnancy test, participants in group A answered that they see this as the task of the GP and not the pharmacist.

S5: “I don’t think it’s their job to say ‘you have to quit smoking’ or whatever. (…) For the pharmacist to say that, no, I don’t think so. If you were to go to the GP, then I think, well, that’s something else. But a pharmacist has nothing to do with that. You’re just getting a pregnancy test, come on. You can also get that at the drugstore.”

In groups B and C the role of the doctor's assistant was also discussed. Just like the pharmacist, doctor's assistants were seen as not qualified and knowledgeable enough to address smoking.

S15: “Doctor’s assistants act like they know everything, while they are not really informed.”

S12: “Not really qualified.”

S8: “I see [doctor’s assistants] as office staff. It sounds strange, but I don't see them as medically trained staff.”

Participants in group B, however, felt that doctor's assistants could provide information about cessation methods, for example, by handing out a brochure.

S10: “To receive a brochure, yes, I like that idea.”

I: “You mean that [the doctor’s assistant] informs you?”

S10: “She has papers lying around. [I would say] like ‘hey, I want to quit smoking, do you have something for me’. (…) Then you don't have to bother the doctor because they are already busy enough.”

Medical specialists, on the other hand, were seen as most qualified to address smoking. Several participants in group A indicated that they would value a quit advice from a medical specialist such as a pulmonologist or cardiologist more than a quit advice from the GP or a nurse.

I: “So, if someone says you have to quit, then..?”

S1: “Yes, not a nurse or whatever. (…) If I'm at the hospital, and the cardiologist or someone says you have to quit, then I think I'll have to accept it, because he knows best.”

I: “And does it also matter if it’s a specialist or a GP [who says you have to quit smoking]?”

S3: “Yes, for me it does.”

I: “Why does that matter?”

S3: “Yes, I think it’s the expertise.”

S5: “A pulmonologist knows [more] about it than the GP.”

## 4 Discussion and conclusion

### 4.1 Discussion

This qualitative study explored the views of people who smoke on the roles of different healthcare providers in smoking cessation care. We mainly included lower-SEP people who smoke, which can be seen as a strength of this study as it can be challenging to involve lower-SEP people in research (15). Notably, the participants in our study perceived medical specialists and GPs to be more qualified to address smoking and provide advice than other types of healthcare providers such as pharmacists and doctor's assistants. This implies that a quit advice from a doctor has more authority than the advice of a non-physician and may thus be taken more seriously by (lower-SEP) people who smoke. A possible explanation is that physicians are generally viewed as authority figures by the public, especially when they wear a white coat (in the literature referred to as “the white-coat effect”) (16). As a result, authority bias may occur where people assume that physicians are more knowledgeable than non-physicians, and are thus more likely to follow the advice of physicians (17).

The participants in this study displayed a limited understanding of the skills and training of non-physicians. For example, doctor's assistants were referred to as “office staff” who may be capable enough to hand out a brochure, while doctor's assistants in the Netherlands are in fact trained to advise on a wide variety of health issues and can also provide several treatments and tests such as wound treatment, cervical screening, blood pressure checks, and electrocardiogram testing (18). Likewise, a pharmacist was merely seen as a store employee who is not qualified enough to provide advice on quitting smoking. Previous studies conducted in the UK concluded that pharmacist-led services, including smoking cessation support, are undermined by a lack of trust from the public (19, 20). Thus, it appears necessary to increase public trust in non-physicians' expertise in smoking cessation care.

Our findings are in line with previous findings showing that the GP is an important source for lower-SEP people who smoke, to hear about and be referred to cessation support (9). Our study shows that lower-SEP people who smoke expect the GP to proactively address smoking and inform them about options to quit. Interestingly, the latter was also mentioned by participants who initially stated that they do not want professional help with quitting, indicating that a lack of perceived need for professional help does not directly mean that the person knows which options are available. In fact, previous research found that people who smoke often do not know which options are available (21–23). Also, previous research demonstrated a strong relationship between hearing about evidence-based cessation support from a healthcare professional and receiving that support during a quit attempt (24). Thus, informing people who smoke about evidence-based cessation support may help to increase their perceived need for professional help.

A few participants mentioned that they only perceive it is appropriate for the GP to address smoking if there is a medical reason to do so. Previous research found that GPs may also not find it appropriate to address smoking if the patient's complaint is not related to smoking (25). Considering that smoking affects nearly every organ in the body, and seriously increases a person's risk of disease and premature death, one can argue that there is always a medical reason to address smoking (26). Patients without smoking-related complaints may need to hear this information when the GP addresses smoking, and advisable would be to mention a specific medical reason when doing so.

### 4.2 Practice implications

This study suggests that there are several opportunities for the delivery of smoking cessation care in practice. First, doctors can use their authority to more often address smoking and provide advice, without fear of harming the patient-provider relationship. Brief interventions, such as the Very Brief Advice approach, exist which doctors can use to address smoking in a non-confrontational manner and directly inform patients about professional help (27). Second, specifically regarding the GP, lower-SEP people who smoke prefer a non-judgmental approach in which the GP asks open questions and allows the person to choose which option they want. This also means that GPs should know which cessation options are available, which may not always be the case (28). In addition, several participants indicated that they would like to read about different options at home, for example on a website, before they make a decision. This was also mentioned by lower educated participants, indicating that the GP should be familiar with websites that are suitable for people with a low level of education or low health literacy.

This study also reveals an important barrier for practice. According to the Dutch Tobacco Treatment Standard of Care (in Dutch: Zorgstandaard Tabaksverslaving), each healthcare provider should at least be able to identify patients who smoke, advise patients who smoke to quit, and refer patients who smoke to evidence based support (29). This includes non-physicians such as pharmacists and doctor's assistants. However, a quit advice and referral by non-physicians may not be as successful or well received if people who smoke do not perceive them to be qualified. To improve people's perceptions of their credibility, non-physicians may need to mention in the conversation that they are knowledgeable on the subject before providing advice. Another possible action is to promote clear and positive messages in the media regarding the expertise of non-physicians (20). GPs could also contribute positive messages on this topic to reinforce public understanding (19, 20). More research is needed to evaluate which actions successfully improve people's perceptions.

### 4.3 Limitations

A few limitations of this study should be addressed. First, the focus groups were conducted in 2019, meaning that the perceptions of people who smoke may have changed in the following years, for example due to the COVID-19 pandemic. Additional research is, however, needed to confirm this. Second, we were mostly able to recruit adults who were over 50 years old. It is possible that older people who smoke have more traditional ideas about the role of healthcare providers compared to younger people who smoke. More research on the perceptions of younger people who smoke is therefore recommended. On the other hand, our sample of mostly older, long-term people who smoke can also be seen as a strength, since they are generally more experienced with quitting smoking and have more often been exposed to healthcare providers than younger people who smoke. Third, the measurement of SEP could have been more comprehensive. While educational attainment is generally a strong indicator of SEP and we also asked participants whether they had a paid job, a more detailed assessment including (household) income or additional indicators might have ensured more precise group classification. Fourth, the data does not allow us to draw conclusions on possible differences in perceptions between lower-SEP and higher-SEP people who smoke, as the focus groups consisted mostly of lower-SEP participants and we did not separate lower-SEP participants from higher-SEP participants. Relatedly, as is inherent to qualitative research, we aimed to gain an in-depth understanding of participants' perceptions, rather than to obtain generalizable findings.

### 4.4 Conclusions

Lower-SEP people who smoke appear to value the advice of a doctor and expect the GP to actively address smoking and provide information on which options are available for quitting. However, non-physicians such as pharmacists and doctor's assistants seem to be perceived to be insufficiently qualified to address smoking. Findings suggest that actions are needed to improve people's perceptions of the credibility of non-physicians in smoking cessation care.

## Data Availability

The datasets used and/or analyzed during the current study are available from the corresponding author on reasonable request.

## References

[1] Reitsma MB Kendrick PJ Ababneh E Abbafati C Abbasi-Kangevari M Abdoli A Spatial, temporal, and demographic patterns in prevalence of smoking tobacco use and attributable disease burden in 204 countries and territories, 1990–2019: a systematic analysis from the Global Burden of Disease Study 2019. Lancet. (2021) 397(10292):2337–60. 10.1016/S0140-6736(21)01169-7 34051883 PMC8223261

[2] Casetta B Videla AJ Bardach A Morello P Soto N Lee K Association between cigarette smoking prevalence and income level: a systematic review and meta-analysis. Nicotine Tob Res. (2017) 19(12):1401–7. 10.1093/ntr/ntw266 27679607

[3] Reid JL Hammond D Boudreau C Fong GT Siahpush M. Socioeconomic disparities in quit intentions, quit attempts, and smoking abstinence among smokers in four western countries: findings from the international tobacco control four country survey. Nicotine Tob Res. (2010) 12(Suppl 1):S20. 10.1093/ntr/ntq051 20889477 PMC2948137

[4] van Wijk EC Landais LL Harting J. Understanding the multitude of barriers that prevent smokers in lower socioeconomic groups from accessing smoking cessation support: a literature review. Prev Med. (2019) 123:143–51. 10.1016/j.ypmed.2019.03.029 30902700

[5] Grech J Sammut R Buontempo MB Vassallo P Calleja N. Brief tobacco cessation interventions: practices, opinions, and attitudes of healthcare professionals. Tob Prev Cessat. (2020) 6:48. 10.18332/tpc/125353 32954061 PMC7493645

[6] Thy T Boker T Gallefoss F Bakke PS. Hospital doctors’ attitudes toward giving their patients smoking cessation help. Clin Respir J. (2007) 1(1):30–6. 10.1111/j.1752-699X.2007.00005.x 20298275

[7] Meijer E Kampman M Geisler MS Chavannes NH. “It’s on everyone’s plate”: a qualitative study into physicians’ perceptions of responsibility for smoking cessation. Subst Abus Treat Prev Policy. (2018) 13(1):48. 10.1186/s13011-018-0186-x PMC6290505 30541580

[8] Meijer E Van Der Kleij RMJJ Chavannes NH. Facilitating smoking cessation in patients who smoke: a large-scale cross-sectional comparison of fourteen groups of healthcare providers. BMC Health Serv Res. (2019) 19(1):750. 10.1186/s12913-019-4527-x 31653215 PMC6815021

[9] Benson FE Nierkens V Willemsen MC Stronks K. Smoking cessation behavioural therapy in disadvantaged neighbourhoods: an explorative analysis of recruitment channels. Subst Abus Treat Prev Policy. (2015) 10(1):28. 10.1186/s13011-015-0024-3 PMC4521474 26227135

[10] Smith AL Carter SM Chapman S Dunlop SM Freeman B. Why do smokers try to quit without medication or counselling? A qualitative study with ex-smokers. BMJ Open. (2015) 5(4):e007301. 10.1136/bmjopen-2014-007301 25933811 PMC4420973

[11] Morphett K Partridge B Gartner C Carter A Hall W. Why don’t smokers want help to quit? A qualitative study of smokers’ attitudes towards assisted vs. unassisted quitting. Int J Environ Res Public Health. (2015) 12(6):6591–607. 10.3390/ijerph120606591 26068089 PMC4483718

[12] Kitzinger J. The methodology of focus groups: the importance of interaction between research participants. Sociol Health Illn. (1994) 16(1):103–21. 10.1111/1467-9566.ep11347023

[13] Krueger RA Casey MA. Focus Groups: A Practical Guide for Applied Research. 5th edn. Thousand Oaks, California: SAGE (2015).

[14] Erlingsson C Brysiewicz P. A hands-on guide to doing content analysis. Afr J Emerg Med. (2017) 7(3):93–9. 10.1016/j.afjem.2017.08.001 30456117 PMC6234169

[15] Emery LF Silverman DM Carey RM. Conducting research with people in lower-socioeconomic-status contexts. Adv Methods Pract Psychol Sci. (2023) 6(4):1–13. 10.1177/25152459231193044

[16] Brase GL Richmond J. The white–coat effect: physician attire and perceived authority, friendliness, and attractiveness. J Appl Soc Psychol. (2004) 34(12):2469–81. 10.1111/j.1559-1816.2004.tb01987.x

[17] Juárez Ramos V. Analyzing the Role of Cognitive Biases in the Decision-Making Process. Hershey, Pennsylvania: IGI Global (2019).

[18] NVDA. Beroepscompetentieprofiel Doktersassistent (BCP) (2019). Available online at: https://www.nvda.nl/themas/beroepscompetentieprofiel (Accessed April 17, 2024).

[19] Gidman W Ward P McGregor L. Understanding public trust in services provided by community pharmacists relative to those provided by general practitioners: a qualitative study. BMJ Open. (2012) 2(3):e000939. 10.1136/bmjopen-2012-000939 22586286 PMC3358628

[20] Greenhalgh T Macfarlane F Steed L Walton R. What works for whom in pharmacist-led smoking cessation support: realist review. BMC Med. (2016) 14(1):209. 10.1186/s12916-016-0749-5 27978837 PMC5159995

[21] Hammond D McDonald PW Fong GT Borland R. Do smokers know how to quit? Knowledge and perceived effectiveness of cessation assistance as predictors of cessation behaviour. Addiction. (2004) 99(8):1042–8. 10.1111/j.1360-0443.2004.00754.x 15265101

[22] Willems RA Willemsen MC Smit ES Nagelhout GE Janssen E de vries H. Which smoking cessation aids are proven effective according to smokers who want to quit smoking? A report from The Netherlands. Tob Control. (2014) 23(6):525–6. 10.1136/tobaccocontrol-2013-051076 23878168

[23] Van Rossem C Spigt MG Kleijsen JRC Hendricx M Van Schayck CP Kotz D. Smoking cessation in primary care: exploration of barriers and solutions in current daily practice from the perspective of smokers and healthcare professionals. Eur J Gen Pract. (2015) 21(2):111–7. 10.3109/13814788.2014.990881 25649048

[24] Van Westen-Lagerweij NA Bommelé J Willemsen MC Croes EA. Mentioning smoking cessation assistance during healthcare consultations matters: findings from Dutch survey research. Eur J Public Health. (2022) 32(5):747–52. 10.1093/eurpub/ckac106 36001051 PMC9527971

[25] van Westen-Lagerweij NA Willemsen MC Croes EA Chavannes NH Meijer E. Implementation of ask-advise-connect for smoking cessation in Dutch general practice during the COVID-19 pandemic: a mixed-methods evaluation using the CFIR framework. Subst Abus Treat Prev Policy. (2023) 18(1):26. 10.1186/s13011-023-00535-0 PMC10169166 37161574

[26] National Center for Chronic Disease Prevention and Health Promotion (US) Office on Smoking and Health. The Health Consequences of Smoking—50 Years of Progress. Atlanta (GA): Centers for Disease Control and Prevention (US) (2014).

[27] van Schayck OCP Bindels L Nijs A van Engelen B van den Bosch A Muller IS The experience of general practitioners with very brief advice in the treatment of tobacco addiction. NPJ Prim Care Respir Med. (2020) 30(1):40. 10.1038/s41533-020-00200-0 32968065 PMC7511299

[28] Van Westen-lagerweij NA Meeuwsen EG Croes EA Meijer E Chavannes NH Willemsen MC. The referral of patients to smoking cessation counselling : perceptions and experiences of healthcare providers in general practice. BMC Health Serv Res. (2021) 21(1):583. 10.1186/s12913-021-06618-7 34140004 PMC8210508

[29] Kerngroep Zorgstandaard Tabaksverslaving. Zorgstandaard Tabaksverslaving 2022 (2022). Available online at: https://www.trimbos.nl/aanbod/webwinkel/af2096-zorgstandaard-tabaksverslaving-2022/ (Accessed April 17, 2024).

