# Supplementary material for: Identifying roles in smoking cessation care for different types of healthcare providers: a qualitative study with people who smoke
Source: Front Health Serv. 2025 Apr 14;5:1421429. doi: 10.3389/frhs.2025.1421429 (PMC12034643; doi:10.3389/frhs.2025.1421429)
Supplement: Supplementary file 1 [file Datasheet1.docx]

Supplementary Material

Identifying roles in smoking cessation care for different types of healthcare providers: A qualitative study with people who smoke

**Naomi A. van Westen-Lagerweij*, Elisabeth G. Meeuwsen, Esther A. Croes, Niels H. Chavannes, Eline Meijer**

*** Correspondence:** Naomi A. van Westen-Lagerweij, NLagerweij@trimbos.nl

Focus group questions

*Only the questions relevant for this study are presented here. Demographic questions were asked in a separate questionnaire.*

1. Do you have a need for professional help from a healthcare provider to quit smoking? Why or why not?
   1. If yes: Which healthcare provider would you go to? What kind of help would you like to receive?
   2. If no: How would you then try to quit smoking?
2. With regard to the GP/practice nurse/doctor’s assistant:
   1. Do you think it is their role to bring up the topic of quitting smoking?
      1. If yes: When do you want them to bring up the topic of quitting smoking?
      2. If no: Why not?
   2. Do you think it is their role to motivate smokers to quit smoking? Why or why not?
   3. Do you think the healthcare provider should choose a treatment for the smoker, or should the smoker do this together with the healthcare provider? Why?
3. With regard to the pharmacist:
   1. Do you think it is their role to give smokers advice on quitting smoking? Why or why not?
   2. Do you think it is their role to refer smokers to the GP practice? Why or why not?
